# Community Intervention System: COVID-19 Control in Inner Mongolia Autonomous Region, China

**DOI:** 10.3390/ijerph182312857

**Published:** 2021-12-06

**Authors:** Yafeng Zou, Qi Wang, Min Deng, Yujie Wang

**Affiliations:** 1 School of Public Administration, Inner Mongolia University, Hohhot 010070, China; vangw7@163.com (Q.W.); wj2579877226@163.com (Y.W.); 2 Faculty of Geographical Science, Beijing Normal University, Beijing 100875, China; 3 Institute of Geographic Sciences and Natural Resources Research, CAS, Beijing 100101, China; Dengmnm@163.com

**Keywords:** community intervention, major public health events, COVID-19 prevention, inner mongolia autonomous region

## Abstract

The COVID-19 epidemic has caused giant influences on people’s life, and China’s communities play an important role in dealing with these major public health events (MPHEs). Community as the grassroots autonomous organization has various significant functions in intervening in MPHEs. The community intervention follows a system which directly influences the anti-epidemic effectiveness. To explore the mechanism, we devise a theoretical system for community intervention, mainly consisting of “organizational structure”, “functional performance” and “internal and external connections”. Questionnaire surveys, the chi-square test, the independent sample T-test, and principal component analysis are used to identify the characteristics of Inner Mongolia Autonomous Region’s (Inner Mongolia) community intervention. Through the empirical research, it is verified that the community intervention in MPHEs is the combination of “the structural response of the organization”, “the performance of the community’s own function”, and “the establishment of internal and external connections”. The central Inner Mongolia delivers the best performance in community intervention compared to eastern Inner Mongolia and western Inner Mongolia. The urban communities commonly perform better than that in the agricultural and pastoral areas. The built system and findings could provide a guidance for future community to improve its intervention capability.

## 1. Introduction

Major Public health emergencies (MPHEs) include outbreaks of major infectious diseases, diseases of unknown causes, large-scale food poisoning, and other sudden events that threaten public health [1]. Such events can cause negative public opinion about administration [2], mass panic [3], shortage of daily necessities supplies [4], and crises of social trust [5]. As a major component of society, the community bears the brunt of MPHEs. Due to its functions of management, service, guarantee of residents’ rights, education, and maintenance of social stability, the community can intervene in response to MPHEs. The government has also encouraged communities to intervene in MPHEs through control and appeal [6], legislative guarantees [7], and cultural construction [8]. In such events, the community uses its functions such as service management, social guarantee, social security [9,10] to reduce the pressure on the government aroused from the citizens.

Research on the community can be roughly divided into ontological research and external research. The former focuses on studying the evolutionary history of community intervention [11], defining concepts related to the community [12], and discussing its nature and orientation [11,13]. Scholars emphasize the importance of community culture, which units the residents [14]. The community’s inner tendencies toward the self-stratification help to optimize its own working pattern [15]. Research on the externalities of the community focuses on discussing the interaction between the community and other organizations [16,17]. The community is the grassroots subject of infiltration of national administrative power [18], thus it has the aim to participate in social affairs. While the community owns interest-driven nature in participation [19], the working process needs to be monitored and guided [20]. The construction of a multi-dimensional evaluation system for community governance and the reasonable working system of the community [21,22] are heatedly discussed.

In China, community is commonly defined as an auxiliary role that supports the operation of a political system. Most international discussions regard the community as a kind of intermediary between different institutions, and the community itself lacks initiative to respond to MPHEs. Both of the ontological research and external research ignore the driving effect of the community’s operation on the operation of external systems, defining which as just a relatively independent individual. The community’s intervention to social system is always seen unconspicuous. Besides, the community’s functional system in an MPHE has not been thoroughly explored, thus the relationship between community intervention and MPHEs lacks discussion.

Empirical research on the emergency working process of community intervention is scant, and thus it is impossible to propose targeted community interventions in MPHEs. Existing research has mostly focused on the macroscopic study area, such as the country and central government, and has not paid sufficient attention to the characteristics of local community intervention. For these reasons, we formulated the following research question: How do communities intervene in the MPHEs?

To answer the question above, this article addressed the practical way of community intervention by offering a built community intervention system mainly composed of three main components as well as latent variables. This system was shaped by the support from existing documentation. Then, this article conducted an empirical research in Inner Mongolia Autonomous Region (hereinafter referred to as Inner Mongolia). We used a questionnaire to analyze the effectiveness and differences of community intervention in MPHEs from the perspective of regions and urban and agricultural and pastoral areas. Finally, the article combined the empirical research with the built system to further point out the detailed and practical way in community intervention, and to manifest the future community’s promotion direction.

## 2. Materials and Methods

### 2.1. Study Area

**Figure 1:**
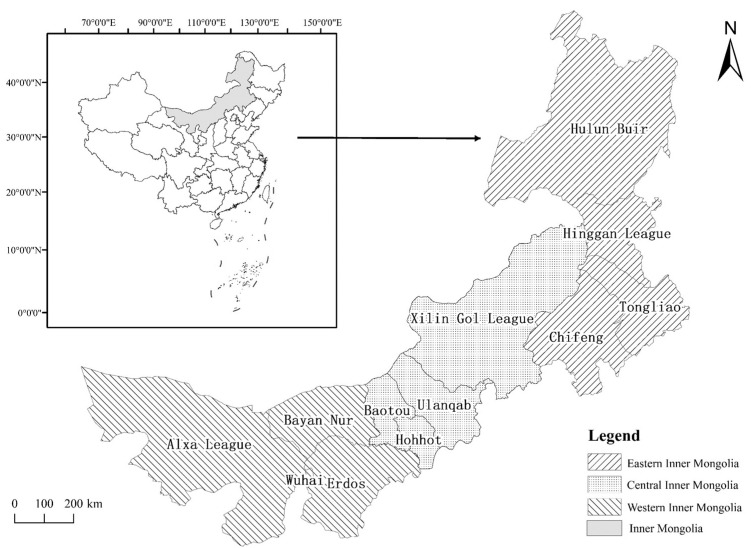
Regional map of the Eastern, Central, and Western Inner Mongolia.

Inner Mongolia (an autonomous region of northern China) has a total area of 1.183 million square kilometers and a population of 25.396 million. In 2020, among the population, the Han nationality accounted for 78.74%, the Mongolian nationality accounted for 17.66%, and the population of other ethnic minorities accounted for 3.60%. Almost each nationality has its own language, but mandarin and Chinese characters together constitute the official language. Inner Mongolia has nine cities and three leagues under its jurisdiction. Due to differences in economic, cultural, and geographic factors, Inner Mongolia is usually divided into eastern Inner Mongolia, central Inner Mongolia, and western Inner Mongolia [23]. The main cities in the eastern Inner Mongolia include Hulun Buir, Hinggan League, Tongliao, and Chifeng; those in the central Inner Mongolia include Xilin Gol League, Ulanqab, Baotou, and Hohhot; and the main cities in the western Inner Mongolia include Erdos, Bayan Nur, Wuhai, and Alxa League (Figure 1). Since the first case of COVID-19 was discovered in Manzhouli (a city with independent planning in Inner Mongolia) on 23 January 2020, many cases have been identified in various cities in Inner Mongolia.

Inner Mongolia took measures immediately by imposing strict restrictions on market supervision, transportation, health supervision, and the movement of people. The government mobilized all sectors of society to participate in responding to the epidemic. Communities in Inner Mongolia also intervened.

Generally, the administrative hierarchy of community governance from top to bottom is the state council, province, city, district (county), street (town), and community. The community is mainly guided by street office in this hierarchical order. When epidemic occurs, in order to uniformly instruct epidemic prevention and control work, epidemic prevention and control headquarters (or leading groups) will be set up at all levels from the state council to street. And then the community intervention is simultaneously directly guided by the street epidemic prevention and control headquarter. The Party Committee and Government of Inner Mongolia implemented the recommendations of “Notice on Strengthening the Community Prevention and Control of Pneumonia Epidemic Caused by New Coronavirus Infection”, “Notice of the General Office of the National Health Commission on the Issuance of the Novel Coronavirus Prevention and Control Plan (Third Edition)”, and the recommendations of other documents. Fighting against the epidemic is a comprehensive task that requires multi-organizational division of work and cooperation. The information of these documents is promulgated as well as transformed into social media information, text messages, etc., by the government to guide each organization and individual to prepare for resisting epidemic. Local communities responded quickly from 24 January on. All tasks were implemented in accordance with the unified deployment of national epidemic prevention and control work. For example, local communities made great efforts to monitor and warn in advance to the residents. They also established advanced data support system, and used grid management, nucleic acid testing, and carpet screening. Once there was any resource shortage or emergency, communities would contact the upper-level organization, and the upper-level organization would dispatch supplies and personnel to deal with the event. Besides, upper leadership and inspection team would also check the prevention situation and give guidance to the community. These actions helped to quickly identify people having close contacts with the infected and accurately delineate prevention and control units.

While ensuring the epidemic is under control in urban and in agricultural and pastoral areas, Inner Mongolia has provided a large number of epidemic prevention materials and COVID-19 manuals to 12 countries, like Russia and Mongolia. Regional work and international cooperation in combating the epidemic have achieved good results. The anti-epidemic experience of Inner Mongolia is of great reference value to the anti-epidemic practice of inland provinces and other countries.

### 2.2. Community Intervention System

Research on community intervention primarily regards the community as the carrier of the governance of social events [24]. Due to this feature, Scholars argue the community has the function of connecting upper- and lower-level organizations in the process of governance [25]. It is also a place where residents live and have emotional interactions with each other [26].

Research focuses on the establishment of a joint structure of governance between communities and multi-party social entities [16,17]. In the community governance system, the core of community’s working should be the subjectivity of people’s governance [27]. The community’s self-evolution also promotes the downward shift of governance in a political system [28].

Some scholars have also discussed the impact of improvements in planning for grassroots governance on the effectiveness of community governance [29], claiming that the current “fragmentation” of social governance needs to be resolved through more reasonable planning [30].

Under the epidemic, the community intervention problems exposed in common include: The measure effects differ from one community to another [31]; low trust causes public panic [32]; rural people have more difficulties in receiving social support [33]; infection risk within the community and inadequate necessities may worsen mental health [34], etc. These various problems threaten the normal operation of community and the residents. To deal with the impacts caused by MPHEs, community needs to explore a suitable working system considering each aspect [32]. Existing researches show that community governance highlights the tendency of structural optimization in political and social systems, focusing on the expansion of its own functions and the enhancement of participatory efficiency. The linkages with various social organizations also necessarily need to be enhanced.

**Figure 2:**
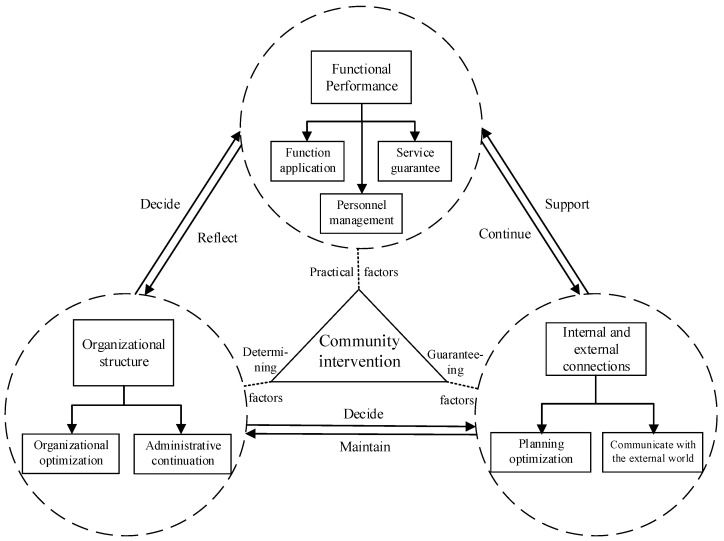
System of community intervention in major public health events (MPHEs).

To refine the core elements of community intervention, this article divides the set of internal elements of community participation and community governance into three main components: “organizational structure”, “functional performance”, and “internal and external connections”. Here, we give definitions to each component: “Organizational structure” refers to a carrier where various resources and participating parts get connected and classified. The existing aim of “organizational structure” is through realizing form optimization and reasonable connection to provide the community itself as well as residents living inside a firm resource allocator; “functional performance” means the community plays its part in serving residents by focusing on different aspects of an event using specific methods and solutions. Usually, the community plays its function from keeping basic function working, staff working quality guaranteeing and thorough services offering to all residents; “internal and external connections” is a non-independent characteristic of the community, which offers the community a platform to improves its learning and growing ability from both inside and outside environment. A community’s response to its emergency management combines “the structural response of organization”, “the performance of the community’s own function,” and “the establishment of internal and external connections” (Figure 2). The organizational structure of community is the decisive factor in community intervention that determines the quality of community’s functional performance, as well as the establishment of internal and external connections. Functional performance is a practical factor, and the implementation of organizational orders broadens internal and external communication channels. Internal and external connections are guarantee factors that ensure the establishment of organizational credibility and normal functioning.

Specifically, at the level of organizational structure, the upper and lower structures determine the efficiency and quality of emergency response functions of community. Emergency organization, with the government as its core, first generates intervention-related orders and issues them to the community, and internal community organizations make emergency adjustments to resist external shocks. The openness of the organizational structure determines the interoperability and common governance of the organization when working internally and externally. An open and efficient organization effectively contacts all resources in society to improve the efficiency of the emergency response. The quality of the functioning of the community reflects its efficiency and structural rationality. In emergencies, the community conducts internal governance from three perspectives—personnel management, functional effectiveness, and service guarantee—to ensure the normal operation of the organization. At the same time, the continuous objective of function is to contact the organizations inside and outside of the community, mobilizing external forces to participate in the internal treatment of MPHEs, as well as improving the operability and continuity of its function. The city epidemic prevention and control headquarter determines the time and approaches of resisting the epidemic, as well as the distribution of personnel and supplies according to both the local epidemic situation and lower-level organization’s feedback. From an external perspective, a reasonable communication and cooperation mechanism between organizational systems is established to promote organizational stability and normal operation. Internal and external connections are also an external guarantee that helps to maintain the organization being open for self-adjustment and spatial optimization. Coordinated intervention and feedback mechanisms are established through optimized planning and resource provision to promote functional performance and optimization. The detailed contents of each latent variables are as follows:

For organizational structure: (1) The community contacts the upper and lower levels of governance, as administration continues at the grassroots level. The outbreak of MPHEs interferes with government administration. To deal with an MPHE, the government first issues requirements and orders to deal with a crisis. The stable implementation of such requirements and orders by the community helps to maintain the stability of the administrative system and helps citizens to fulfill their social duty. (2) The community is responsible for organizational control and structural upgrade. It has independent departments for decision making, the appointment of personnel, work distribution, material scheduling, and financial management. The upgrade and replacement of various departments and internal personnel constitute the overall structural optimization in the community intervention. When MPHEs occur, the community can optimize its organizational management and control, reorganize its structure, and pool its resources to resist the impact of risks.

For functional performance: (1) The community has a rich variety of functions and can fully intervene in MPHEs—it encompasses all aspects of its residents’ lives. The outbreak of MPHEs restricts people’s travel, living, entertainment, and educational activities. The coverage of community functions coincides with the impact of MPHEs. When the community exerts its initiative, it helps to reduce the impact of MPHEs on people’s lives. (2) The community is responsible for personnel management, which is the core of intervention. People are the direct objects of the harm caused by MPHEs and the core driving force for the community management. Because of the unique emotional bond and important relationship between the community and the residents, when an MPHE threatens the safety of people, the community acts quickly and the residents can be mobilized into defending MPHEs under crisis management. (3) The community provides services and protects human rights; its service-related attributes enable the community to understand people’s needs. The community can guarantee social equality during a crisis and help protect the rights of vulnerable groups.

For internal and external connections: (1) The community communicates with the external world. It belongs to the system of social organization, and is inseparable from social affairs. It provides external information and resources to residents to build a bridge between them and society. (2) Communities are in densely populated areas and are the first to be impacted by MPHEs. The community has the tendency to assess, upgrade, and optimize its planning; and it participates in the transformation, development, and governance of the environment, which builds the community and the community itself practices that as well.

### 2.3. Research Ideas

Community intervention has its own characteristics and unique importance at the grassroots level. A questionnaire was designed for this article using a five-point Likert scale (1 = very inconsistent, 2 = inconsistent, 3 = uncertain, 4 = consistent, 5 = very consistent) [35]. Based on the system of community intervention and various reports, such as “Notice on Strengthening the Community Prevention and Control of Pneumonia Epidemic Caused by New Coronavirus Infection”, “Notice of the General Office of the National Health Commission on the Issuance of the Novel Coronavirus Prevention and Control Plan (Third Edition),” as well as the respective economic and cultural characteristics of urban and of agricultural and pastoral areas in Inner Mongolia, the questionnaire covered the three laten variables of organizational structure, functional performance, and internal and external connections. The data obtained were processed using Statistical Product and Service Solutions24.0. (SPSS).

**Figure 3:**
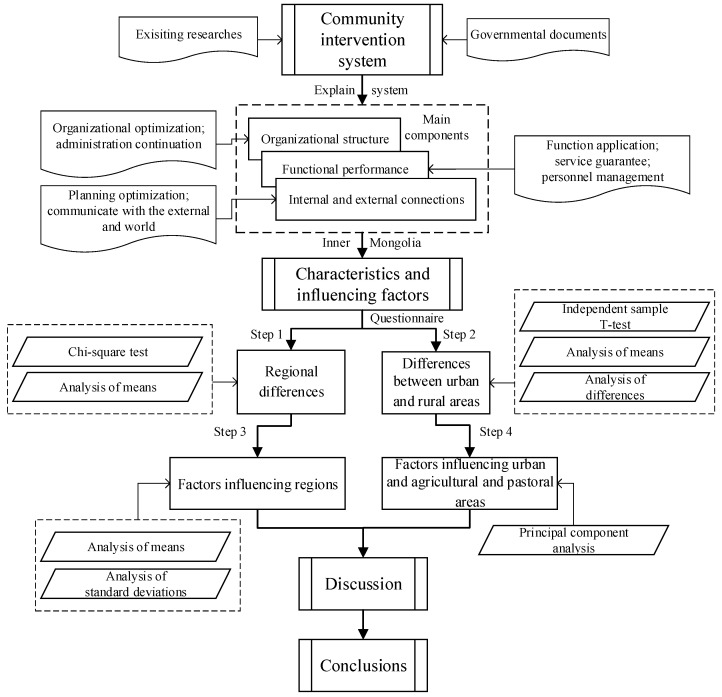
Flowchart of research.

To analyze the data, we first used the chi-square test to identify differences among the eastern, central, and western Inner Mongolia regarding the latent variables of community intervention. An independent sample T-test was used to analyze the differences in the latent variables between urban, and agricultural and pastoral areas. The means and standard deviations of the scores of the questionnaire were combined to explore reasons for the differences in regional community intervention. For the differences between urban and rural areas, principal component analysis was used. And differences in community intervention among regional, urban, and agricultural areas, as well as factors influencing the differences, were summarized (Figure 3).

### 2.4. Data Sources and Questionnaire Design

The design of the questionnaire followed the established system of community intervention in MPHEs. The organizational structure, functional performance, and internal and external connections of community intervention were set as latent variables. Specific variables were selected and classified according to the epidemic prevention measures of the communities of Inner Mongolia. For this selection, news reports from Chinese mainstream media, such as from the People’s Daily, Sohu News, and Sina News, as well as special reports and relevant documents from the website of the government of Inner Mongolia were used. The choice of the specific variables for organizational structure mainly considers the degree of coordination between the community and the external organizational system, the extent of the organization’s own initiative, and the outward effects of internal and external organizational systems and their potential drawbacks while working (X_1_–X_7_). Each specific variable for functional performance was selected from the categories of intensity, effects, and deficiencies of measures taken by the community for epidemic prevention (X_8_–X_14_). The variables for internal and external connections were selected by considering the community’s own sociality and its internal and external interoperability in terms of planning and personnel (X_15_–_20_) (Table 1).

To understand the intervention-related behaviors of the Inner Mongolia community for epidemic prevention, a random sampling method was adopted. From 20 April to 29 June 2020, people from nine cities and three leagues (mainly residents and community workers) in Inner Mongolia filled out questionnaires in Chinese online anonymously. A total of 2500 questionnaires were distributed, of which 2367 were valid for a success rate of 94.68%. The subjects in this survey included young (18–45 years old), middle-aged (46–69 years old), and older people (over 69 years old) (Table 2). The number of groups was relatively evenly distributed. Part of the data was supplemented through field visits to several villages in different regions for later explaining the reason why agricultural and pastoral areas commonly had a lower score compared to urban areas. The Cronbach’s alpha was 0.960 (>0.7), indicating that the data were credible [36]. The Kaiser–Meyer–Olkin measure of sampling adequacy was 0.950 (>0.6), and *p*-value of Bartlett’s test of sphericity was 0.000 (<0.05), indicating that the data of the questionnaire was of good quality [37].

## 3. Results

### 3.1. Differences in Community Intervention

#### 3.1.1. Regional Differences

Pearson’s chi-square test was used for the three latent variables mentioned above and the overall score, and *p*-value of each variable was less than 0.05, which means there were obvious differences in the overall scores of the variables among regions [38] (Table 3).

We calculated the average scores for the eastern, central, and western Inner Mongolia in terms of three latent variables (Table 3). Significant regional differences in scores for community intervention, organizational structure, functional performance, and internal and external connections were observed. The central Inner Mongolia delivered the best performance in the ensemble of community intervention, followed by the eastern and western Inner Mongolia. Scores of the central Inner Mongolia were significantly better than those of the eastern and western Inner Mongolia for all latent variables. The scores of the western Inner Mongolia for three latent variables were lowest compared to the other two regions. For the whole Inner Mongolia, the overall situation was much worse than that in the central Inner Mongolia, which had the highest scores, and was better than that in the western Inner Mongolia. The whole Inner Mongolia’s scores for organizational structure were close to those for functional performance. This indicated that the overall community intervention there had prioritized organizational structure and functional performance; this also indicated that the internal and external connections played a supporting role for the former two working.

#### 3.1.2. Differences between Cities and Agricultural and Pastoral Areas

An independent sample T-test was conducted on the three latent variables. According to existing studies, a *p*-value less than 0.05 means the difference between each variable is significant [39]. Significant differences were found in the effects of intervention between cities and rural areas in the eastern, central, western and the whole Inner Mongolia, with a *p*-value of less than 0.05. The mean values of and differences in the latent variables of community intervention and their overall effects were relatively uniform and stable within each region (Table 4). On the whole, each and the overall average scores of latent variables of urban community intervention had a spatial distribution that decreased from east to west. That is, the scores of eastern Inner Mongolia were slightly better than the central Inner Mongolia and significantly better than the western Inner Mongolia. The urban community intervention scores of Inner Mongolia were roughly the same as those of the central Inner Mongolia. The latent variables and overall scores in community intervention of the central Inner Mongolia’s agricultural and pastoral areas were better than those of the eastern and western Inner Mongolia; and those of the western Inner Mongolia were significantly lower than those of the central and eastern Inner Mongolia. Overall, the values of the latent variables and overall scores of urban community intervention were significantly higher than those of community intervention in agricultural and pastoral areas. The disparities were less significant in the central Inner Mongolia than in the eastern and western Inner Mongolia. The latent variables and overall effect difference in the whole Inner Mongolia were similar to those in the eastern Inner Mongolia.

### 3.2. Factors Affecting Effectiveness of Community Intervention

#### 3.2.1. Factors Influencing Regions

**Figure 4:**
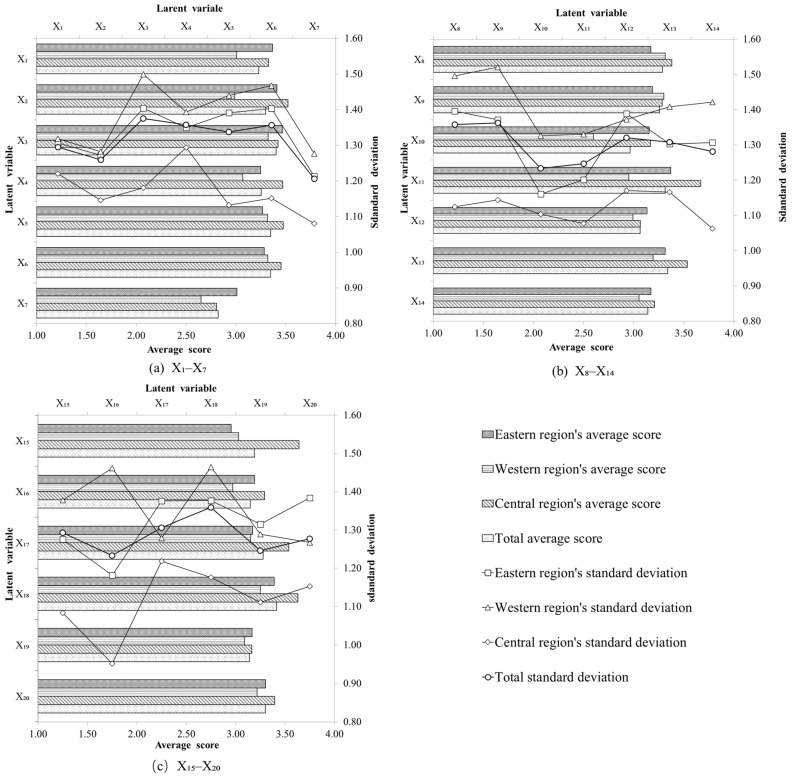
Regional values of latent variables.

In Inner Mongolia, there were obvious regional differences in the latent variables and in the performance of each internal link. The central Inner Mongolia delivered an outstanding performance and the western Inner Mongolia had a poor performance. The epidemic prevention behaviors and corresponding links have not been effectively unified between each region and within each region of Inner Mongolia (Figure 4). Organizational structure and functional performance had a great impact on the anti-epidemic effect of a region. However, the functional performance of a region was relatively weaker than its organizational structure and internal and external connections; and the diversity of helping methods for residents and the mobilization of and participation in anti-epidemic efforts needed to be improved. Each variable’s standard deviation of the central Inner Mongolia was lower than that of the eastern and western Inner Mongolia and that of east was better than the west. It suggests that the internal consistency of the community intervention of central Inner Mongolia was better than that of eastern Inner Mongolia. Also, the inner continuity of eastern Inner Mongolia was better than that of western Inner Mongolia. We also found that cities with infected people in the same region focused on management and control, while that of the other cities was relatively weak. There were obvious differences in implementation among regions and within regions.

The regional distribution of the specific variables is shown in Figure 4. The scores of specific variables in the eastern Inner Mongolia mainly reflected the reasonable construction and efficient operation of organization and the key role of community functions. For example, the eastern Inner Mongolia did well in the quarantine of people, mobility control, and psychological counseling. The scores of specific variables in the central Inner Mongolia mainly reflected efficient cooperation between the upper and lower organizations, efficient function operation, and active cooperation with the outside world. Mainly because the central Inner Mongolia has made great efforts to build smart cities, owning great administrative effectiveness and strict upper supervision. The scores of specific variables in the western Inner Mongolia mainly reflected the functional operation of organization and the functioning of the community itself. However, the western Inner Mongolia’s community also had insufficient manpower, ineffective implementation, and a relatively weak organizational structure. The score of variable X_7_ in all regions was generally low, meaning the community’s anti-epidemic work was not transparent, and the implementation was not completely following procedures. Variable X_15_ in the eastern Inner Mongolia and variable X_10_ in the western Inner Mongolia also scored significantly lower than other variables. This indicates that the construction of a 15-min living circle was not highly built in the eastern and western Inner Mongolia, respectively, and that vulnerable groups in the epidemic were not well taken care of. Only in the central Inner Mongolia, were the scores of various variables relatively balanced, with no obvious low score, except for X_7_.

#### 3.2.2. Factors Influencing Urban and Agricultural and Pastoral Areas

Urban Areas; The results of the urban questionnaire were analyzed by principal component analysis [40], where the principal components were extracted using a correlation matrix through the maximum variance method. The maximum number of iterations for convergence was five. Taking the first five principal components with characteristic roots greater than one, the cumulative contribution of the first five principal components was 63.206%; that is, 63.206% of all the information could be explained by the first five principal components (Table 5).

In the principal component factor analysis, to ensure the results convictive, factors with loads smaller than 0.5 need to be deleted. The 20 specific variables in this questionnaire, except X_7_ (0.460), X_16_ (0.484), and X_18_ (0.479), had a common variance of higher than 0.5. Therefore, the five principal components extracted should be able to better explain the important information of the original variables (Table 5).

Of the five principal components, the rising motivation of the self-service component F1 corresponded to the latent variable of the functional performance of community intervention. The community implemented its functions through the diversified development and comprehensive coverage of services within it. The services included the transmission of information, purchase of basic supplies for isolated personnel, help for vulnerable groups, management of community personnel. Also, urban communities mobilized community personnel to participate in epidemic prevention, and made progress in environmental sanitation, the use of intelligent means of service, and cooperation with external organizations. The self-supervision component F2, self-optimization component F3, and efficiency enhancement component F4 corresponded to the latent variable of organizational structure. The rationality and credibility of the organizational system were reflected in its own structural function. For example, the community could improve itself by its self-supervision, self-optimization of the structure, and self-improvements in the efficiency of organizational operation. The effective communication and interaction of the three components constituted a perfect organizational architecture for community intervention. The spatial unity component F5 corresponded to the latent variable for internal and external connections, which worked mainly through controlling over the movement of people and reasonable community planning. According to the data, the load of functional performance (component F1) was 23.119%, that of organizational structure (component F2, F3, and F4) was 32.951%, and that of internal and external connections (component F5) was 7.136%. Thus, community interventions in urban communities primarily focused on optimizing the organizational structure and strengthening the community’s functions. The establishment of internal and external connections serves as supporting points in the urban communities fighting against MPHEs. The effectiveness of the interface between the community and external organizations (X_17_) was classified as self-service component F1. This indicates that the function of community intervention intersected internal and external connections. The influence of internal and external connections was minor because they had not been established effectively.

The community intervention related behavior in cities verified the system of community intervention. In an MPHE, the city had relatively good organizations. The most important consideration was to give full play to community functions, so that the community could deal with the emergency. The urban community has a large population over a vast area of land, and upper-level government cannot provide detailed supervision and control. Thus, there was a greater reliance on the community’s own initiative and its sociality in the system of social organization to optimize control over personnel and the mobilization of resources. Moreover, the urban community’s own enforcement and emergency experience were insufficient, and its working of function needed support from other organizations. Thus, guidance and supervision from upper-level organizations and the effectiveness of the community’s own organizational construction determined the performance of the community functions under the epidemic. Internal and external connections were mainly manifested in the emergency flow of personnel and materials, and in spatial factors. At the beginning and peak of the emergency, the community struggled to resist the damaging impact by itself and often needed external resources. The organizational structure, functional performance, and internal and external connections of the community intervention were interrelated to constitute the core elements of community intervention, enabling which to link with public emergencies.

Agricultural and Pastoral Areas; Principal component analysis was also used on the results of the questionnaire for agricultural and pastoral areas. The principal components were extracted by rotating the correlation matrix using the maximum variance method. The maximum number of iterations was six. The first six principal components with characteristic roots scoring greater than one had a cumulative contribution of 64.612% (Table 6).

Of the 20 specific variables in the questionnaire for agricultural and pastoral areas, except for X_12_ (0.431) and X_14_ (0.483), the common variance of 18 factors was greater than 0.5. This shows that extracting six principal component factors from the remaining 18 variables can explain the important information of the original variables (Table 6).

Of the six principal components, the rising motivation of the optimized supervision component F1 corresponded to the latent variable of organizational structure in the system of community intervention. The community ensured the integrity of the organizational structure and functional effectiveness during the epidemic by improving its emergency departments. It also receives the guidance provided by the upper level in terms of governance. Finally, the community improves its organization’s functions to provide mandatory and service measures for residents. The organizational connection component F2 corresponded to the latent variable of organizational structure. The community improved its organizational effectiveness by giving full play to the autonomy of grassroots communities in agricultural and pastoral areas for epidemic prevention, and by reducing the bureaucracy that was prone to appear. The community optimized its organizational structure to connect all links of implementation of epidemic prevention. The community focused on improving environmental sanitation, meeting the needs of farmers and herdsmen in the 15-min living circle. This experience helped to form a practice model that focused on organizational optimization and connected various functions. The personnel management component F3 corresponded to the latent variable of functional performance. Community interventions carried out in pastoral areas focused on assistance to vulnerable groups, improving the ability of grassroots cadres to perform their duties, and launching grid governance to compensate for the shortage of manpower and loose structures. The community also enhanced the effect of implementation of grassroots community intervention by strengthening the efficiency of people-oriented service and functions. The organizational management component F4 also corresponded to the latent variable of organizational structure. Component F4 strengthened community management and control at the grassroots level to receive and disseminate information in a timely manner to improve the strength and accuracy of execution. The external connection component F5 corresponded to the latent variable of internal and external connections, mainly in the form of effective grassroots communities in farming areas, and in the form of cooperation with external organizations, to mitigate the community’s weak foundations, lack of materials, and shortage of labor. The spatial unity component F6 corresponded to the latent variable of internal and external connections. The internal planning of communities in agricultural and pastoral areas was likely to be incomplete, or to lack space for a public emergency. This is often caused by the high density of buildings and lack of compliance with emergency management needs. To optimize community space in planning involves building a spatial distribution system that connects the inside space of the community with the outside space. The control of personnel flow in agricultural and pastoral areas was affected by the scattered distribution of each residential area and was, therefore, difficult to manage. However, the management of personnel in agricultural and pastoral areas was a very important starting point in dealing with the epidemic. According to the data, the organizational structure (component F1, F2, and F4) accounted for 35.142% of the load; the functional performance (component F3) accounted for 12.691%; and the internal and external connections (component F5 and F6) accounted for 16.779%. Grassroots communities in agricultural and pastoral areas were weak in their own functions, owing to a lack of labor and low administrative efficiency. The community relied on upper-level organizations and outside support. Because the farming and pastoral areas were sparsely populated and difficult to manage, more emphasis was placed on the establishment of effective internal and external connections to ensure the organization’s operation and functioning. However, the overall effectiveness of community intervention in these areas was weak and unable to maintain the integrity and continuity of each link.

## 4. Discussion

The implementation of community-led, anti-epidemic measures in Inner Mongolia shows that community intervention in MPHEs is complex. Responses to MPHEs require inter-organization and intra-organization cooperation. It is essential to give play to the function of the community as the main body of grass-roots governance. At the same time, giving play to the internal and external connections of the organization is an effective way to achieve effective access to material, personnel, and information. Compared to the actual situation, the central Inner Mongolia, which had a stronger resistance to the epidemic, had a high degree of coordination in the three latent variables of community intervention; while the eastern and western Inner Mongolia had a low degree of coordination in these aspects. In urban communities, the coercive roles of organizations and community functions are significant in supporting the fight against the epidemic. However, in agricultural and pastoral areas, due to their own restrictive conditions, the function of organizations was relatively weak, while the community functions and external assistance were stronger.

From the scores of community intervention in both urban areas and agricultural and pastoral areas, the organizational structure usually had a higher score than the other two latent variables. In MPHEs, this system could have advantages in well organizing all resources, especially in dispatching the medical staff, bed resources, medical equipment, etc. In urban areas, the basic emergency-dealing resource is relatively adequate and the community has a relatively mature operation mode thus behaving better than rural area community. Hence, strengthening assistance and improving rural communities’ own organizational structure construction strongly influences a whole area’s community responding efficiency.

It is worth noticing that in the principal component analysis, the urban community’s load of functional performance outweighed much more than that of agricultural and pastoral. This finding shows that equalized community services have not covered both urban and rural areas. This supply difference may further cause rural residents’ low trust in community. To deal with this probable situation, both urban and rural communities should focus on smart community construction, which aims at using various intelligent technologies and methods to enhance and balance the community’s functional performance. Besides, urban communities could share the constructing experience with rural communities. Through offering more humane and balanced services, residents will participate in community governance more actively thus improving the effectiveness of community intervention.

The functions of urban community intervention intersected and intercommunicated with internal and external connections. However, in the actual epidemic prevention practice, the internal and external connections of the community were weaker than the other latent variables because they were commonly not well established. This is a part easily ignored or passively practiced in reality, which in turn may reduce the operating efficiency of other components. In inner Mongolia’s community intervention practice, communities prefer to seek for assistance when emergency occurs, but daily connections with external world are insufficient. Information and supplies are thus not fluently transmitted in urgency.

The above problems revealed have guidance in promoting community construction in the future. Strengthening the community’s own initiative is one of the solutions. Specifically, the community should focus on its intervention system well working. The “organizational structure” should be the leading and starting part of community intervention. Under special circumstances, the government and the community should participate together instead of being led by one only. On the one hand, to increase residents’ life satisfaction, the community could construct more public service facility and residents’ collective learning activity, which is also a kind of “functional performance” improvements. On the other hand, the community could enhance “internal and external connections”, thus in MPHEs, the community could aid and be aided with the outside world quickly. In an MPHE, it is necessary to improve community intervention, implementing a system with a “structural response of the organization”, “performance of its own function”, and “establishment of internal and external connections”. This helps to organically link various measures to enhance the overall effectiveness of community intervention. Efforts must be made in reducing the difference in the effectiveness of the implementation of epidemic prevention measures between urban and rural areas. It is also necessary to improve public awareness, and to increase people’s sense of participation in prevention measures.

Due to the impact of COVID-19, on-site research could not be all conducted, which may cause some inaccuracy in reflecting residents’ true willingness. Inner Mongolia did a relatively good job in fighting against the epidemic, but due to our research team’s understaffed situation, China’s other autonomous regions’ community intervention was not compared with. In the future, the research area could be expanded to larger regions. An on-site and long-period empirical research could be applied to track resident’s precise sense of community intervention.

## 5. Conclusions

As the COVID-19 has caused serious impacts throughout the world, China’s community has played an important role in dealing with the epidemic. By formulating a system for community intervention in the case of an MPHE, this article used a chi-square test to determine the differences and obtain average scores in organizational structure, functional performance, internal and external connections, and in the overall effect of community interventions. Independent sample T-tests were used to examine differences in the scores of the latent variables of urban and rural areas in the eastern, central, western Inner Mongolia and the whole Inner Mongolia. We combined the questionnaire scores to explore reasons for regional differences in urban community intervention, and used principal component analysis to extract and classify the main factors influencing community intervention in urban and agricultural and pastoral areas. The following conclusions can be drawn. (1) The system of community intervention in MPHEs was the combination of “the structural response of the organization”, “the performance of the community’s own function”, and “the establishment of internal and external connections”. The community responds to the epidemic with the three organically integrated and affecting each other. (2) There were significant differences in the scores of organizational structure, functional performance, and internal and external connections among the eastern, central, and western Inner Mongolia. Overall, the score of each latent variable in the central Inner Mongolia was better than the scores in the eastern and western Inner Mongolia. The effective unification of epidemic prevention behaviors within each region and within Inner Mongolia was not achieved. (3) The mean and difference of each latent variable, as well as its overall effect, were relatively uniform and stable within each region. The scores of the city’s three latent variables had a spatial distribution that decreased from eastern Inner Mongolia to western Inner Mongolia. The score gap was generally large, indicating that urban and agricultural and pastoral community interventions have not yet been unified and rendered homogeneous. (4) Urban community intervention focused on optimizing the organizational structure and strengthening the functions of the community, with the establishment of internal and external connections as starting points. Community intervention in agricultural and pastoral areas relied on support from upper-level organizations and the outside world, and the community’s own functions were weak.

## Figures and Tables

**Table 1:** Community intervention questionnaire for Inner Mongolia.

Intent	Latent Variables	Number	Specific Variables
Performances of community intervention in MPHEs in Inner Mongolia	Organizational structure(X_1_–X_7_)	X_1_	Community’s functional efficiency
		X_2_	Community’s ownership of epidemic prevention
		X_3_	Rapid response
		X_4_	Soundness of emergency department
		X_5_	Level of control
		X_6_	Top spot check
		X_7_	Formalism, bureaucracy
	Functional performance(X_8_–X_14_)	X_8_	Information sharing effectiveness
		X_9_	Quarantine and help purchase
		X_10_	Poverty alleviation, key assistance
		X_11_	Personnel management level
		X_12_	Voluntary mobilization
		X_13_	Sanitation improvement
		X_14_	Application of multimedia and internet intelligent service level
	Internal and external connections(X_15_–X_20_)	X_15_	15-min lap convenience
		X_16_	Grid governance
		X_17_	Effectiveness of community and external organization interface
		X_18_	Cooperation between communities and outside organizations
		X_19_	Rationality of community planning
		X_20_	Effectiveness of personnel flow control

**Table 2:** Basic respondent Information.

Item	Category		Number of Copies	Percentage
Gender	Male		1001	42.29%
	Female		1366	57.71%
Age	Youth		932	39.37%
	Middle aged		764	32.28%
	Elderly		671	28.35%
Area	Eastern Inner Mongolia	Urban area	484	20.45%
		Agricultural and pastoral area	330	13.94%
	Central Inner Mongolia	Urban area	451	19.05%
		Agricultural and pastoral area	277	11.70%
	Western Inner Mongolia	Urban area	572	24.17%
		Agricultural and pastoral area	253	10.69%

**Table 3:** Scores of the mean and chi-square test of community intervention in each region.

Latent Variables	Chi-Square Test			Average Score			
	Value	Freedom	*p*-Value	Eastern Inner Mongolia	Western Inner Mongolia	Central Inner Mongolia	The Whole Inner Mongolia
			(Double Side)				
Community intervention	1052.810	156.000	0.000	3.237	3.090	3.372	3.227
Organizational structure	472.000	56.000	0.000	3.294	3.097	3.354	3.244
Functional performance	800.927	56.000	0.000	3.214	3.057	3.330	3.195
Internal and external connections	569.850	48.000	0.000	3.196	3.119	3.444	3.245

**Table 4:** Comparison of latent variables and the overall scores of community intervention among the regions.

Region	Latent Variables	T-Test		Mean		Difference
		T	*p*-Value (Two-Tailed)	Urban Area	Agricultural and Pastoral Area	
Eastern Inner Mongolia	Organizational structure	68.392	0.000	3.832	2.168	1.664
	Functional performance	57.198	0.000	3.831	2.213	1.618
	Internal and external connections	53.634	0.000	3.802	2.132	1.669
	Overall effect	73.282	0.000	3.867	2.157	1.710
Central Inner Mongolia	Organizational structure	43.995	0.000	3.932	2.217	1.715
	Functional performance	32.505	0.000	3.979	2.289	1.690
	Internal and external connections	31.270	0.000	3.882	2.235	1.647
	Overall effect	53.956	0.000	3.936	2.112	1.824
Western Inner Mongolia	Organizational structure	65.980	0.000	3.720	1.665	2.054
	Functional performance	52.778	0.000	3.683	1.772	1.911
	Internal and external connections	55.818	0.000	3.740	1.514	2.226
	Overall effect	59.420	0.000	3.739	1.718	2.021
The whole Inner Mongolia	Organizational structure	40.080	0.000	3.866	2.569	1.296
	Functional performance	31.777	0.000	3.862	2.527	1.335
	Internal and external connections	30.422	0.000	3.793	2.575	1.218
	Overall effect	40.043	0.000	3.955	2.612	1.343

**Table 5:** Results of principle components analysis for the urban areas.

Principal Component	Square Sum of Rotational Loads			Variables with Large Loads	Principal Component	Rising Motivation
	Total	Percentage of Variance	Cumulative Percentage			
F1	4.624	23.119	23.119	X_8_ (0.568), X_9_ (0.513), X_10_ (0.683), X_11_ (0.827), X_12_ (0.722), X_13_ (0.621), X_14_ (0.601), X_17_ (0.753)	Self-service component	Functional performance
F2	2.789	13.945	37.604	X_5_ (0.768), X_6_ (0.750)	Self-supervision component	Organizational structure
F3	1.963	9.814	46.877	X_2_ (0.826), X_4_ (0.748)	Self-optimization component	Organizational structure
F4	1.839	9.193	56.070	X_1_ (0.685), X_3_ (0.566)	Efficiency enhancement component	Organizational structure
F5	1.427	7.136	63.206	X_19_ (0.831), X_20_ (0.740)	Spatial unity component	Internal and external connections

**Table 6:** Results of principle components analysis for the agricultural and pastoral areas.

Principal Component	Square Sum of Rotational Loads			Variables with Large Loads	Principal Component	Rising Motivation
	Total	Percentage of Variance	Cumulative Percentage			
F1	2.640	13.201	13.201	X_4_ (0.808), X_6_ (0.704), X_9_ (0.719)	Optimized supervision component	Organizational structure
F2	2.638	13.19	26.391	X_2_ (0.675), X_3_ (0.503), X_7_ (0.561), X_13_ (0.563), X_15_ (0.739)	Organizational connection component	Organizational structure
F3	2.538	12.691	39.082	X_10_ (0.626), X_11_ (0.818), X_16_ (0.771)	Personnel management component	Functional performance
F4	1.750	8.751	47.833	X_5_ (0.707), X_8_ (0.774)	Organizational management component	Organizational structure
F5	1.709	8.544	56.377	X_17_ (0.855), X_18_ (0.622)	External linkage component	Internal and external connections
F6	1.647	8.235	64.612	X_19_ (0.759), X_20_ (0.821)	Spatial unity component	Internal and external connections

## Data Availability

Not applicable.
